# Armstrong strain lymphocytic choriomeningitis virus infection after accidental laboratory exposure

**DOI:** 10.1186/s12985-023-02258-x

**Published:** 2023-12-12

**Authors:** Laurence Caron, Jean-Sébastien Delisle, James E Strong, Yvon Deschambault, Félix Lombard-Vadnais, Annie-Claude Labbé, Sylvie Lesage

**Affiliations:** 1 Département de microbiologie, infectiologie et immunologie, Université de Montréal, Montréal, QC Canada; 2 Immunologie-oncologie, Centre de Recherche de l’Hôpital Maisonneuve-Rosemont, Montréal, Canada; 3 Département de médecine, Université de Montréal, Montréal, QC Canada; 4 National Microbiology Laboratory, Public Health Agency of Canada, 1015 rue Arlington Street, Winnipeg, MB R3E 3R2 Canada; 5 Department of Pediatrics & Child Health, College of Medicine, Faculty of Health Sciences, University of Manitoba, Winnipeg, MB Canada; 6 Department of Infectious Diseases and Medical Microbiology, College of Medicine, Faculty of Health Sciences, University of Manitoba, Winnipeg, MB Canada; 7 Service de microbiologie et d’infectiologie, Hôpital Maisonneuve-Rosemont, CIUSSS de l’Est-de-l’île-de-Montréal, Montréal, QC Canada

**Keywords:** LCMV, Armstrong, Laboratory accidental exposure

## Abstract

**Background:**

Lymphocytic choriomeningitis virus (LCMV) is a human pathogen naturally present in wild rodents. In addition, LCMV is routinely used in immunology research as a model of viral infection in mice. The Armstrong common laboratory strain and the Clone-13 variant induce acute and chronic infections in mice, respectively. The frequent use of this virus in laboratory settings is associated with a risk of human infection for laboratory personnel. In contrast to LCMV Clone-13, few human laboratory infections with LCMV Armstrong have been reported, leading to a poor understanding of symptoms related to infection with this specific LCMV strain.

**Case presentation:**

A researcher accidentally infected herself percutaneously with LCMV Armstrong. Symptoms including headaches, dizziness, eye pain and nausea appeared seven days post-exposure and lasted ten days. LCMV-IgM antibodies were detected at 28 days post-infection and IgG seroconversion was observed later. Complete recovery was confirmed three months post exposure.

**Conclusions:**

Research involving live viruses comes with the risk of infection for research personnel. This case is the first reported accidental human infection with LCMV Armstrong. The symptoms differed from reported infections with LCMV Clone-13, by the absence of fever and vomiting, and presence of leg numbness. This report will therefore help clinicians and public health authorities to recognize the symptoms associated with LCMV Armstrong infections and to offer appropriate counselling to individuals who accidentally expose themselves.

## Background

Lymphocytic choriomeningitis virus (LCMV) is an enveloped RNA virus that belongs to the *Arenaviridae* family and its natural host and reservoir is the house mouse (*Mus musculus)* [1]. It was discovered in 1933 by Charles Armstrong, in St-Louis, when he studied epidemic encephalitis [2–4]. LCMV estimated seroprevalence in the general population ranges between 2 and 20%, depending on the geographic location [2].

Most human LCMV infections occur through exposure to infected rodent excretions via the respiratory tract of the host, where LCMV replicates, moves to the bloodstream, and invades multiple organs [5]. The virus may eventually reach the brain, more specifically the choroid plexus, the ventricular ependymal linings and leptomeninges, where it can replicate to high titers [6]. Typically, LCMV infections in humans will cause an early wave of acute symptoms, followed by a more severe second wave, one-month post-infection [5, 7, 8].

In research laboratories, the Armstrong LCMV strain is routinely used as prototypical models of viral infections in mice. While the Armstrong strain generates an acute viral infection in mice, the Clone-13 variant, a derivative of the Armstrong strain, causes a more chronic infection [9]. In accidental infections of immunocompetent laboratory personnel with LCMV, about one third of individuals are asymptomatic, while others develop flu-like symptoms [2]. In most severe cases, meningitis and encephalitis have been described [7, 10, 11], with a mortality rate of less than 1% [6]. The long-term effects of LCMV Clone-13 infection are still unclear. To our knowledge, no cases of accidental human LCMV Armstrong infection have been reported, limiting our understanding of symptoms related to infection via the percutaneous route. Here, we report the case of an accidental LCMV Armstrong infection in an immunocompetent laboratory worker.

## Case presentation

In May 2023, a 25-year-old female researcher living in Montreal presented to the emergency department following an accident, as per her workplace requirements for all work-related accidents involving body fluids. While she was injecting LCMV Armstrong into the peritoneal cavity of mice to study antiviral T cell response, she accidentally injected her finger. This percutaneous accident with a needle containing a solution of LCMV Armstrong was her first exposure to the virus. Other than a cerebral venous sinus thrombosis, which occurred in 2015, her past medical history was unremarkable, with no medical condition causing potential immunosuppression.

**Fig. 1:**
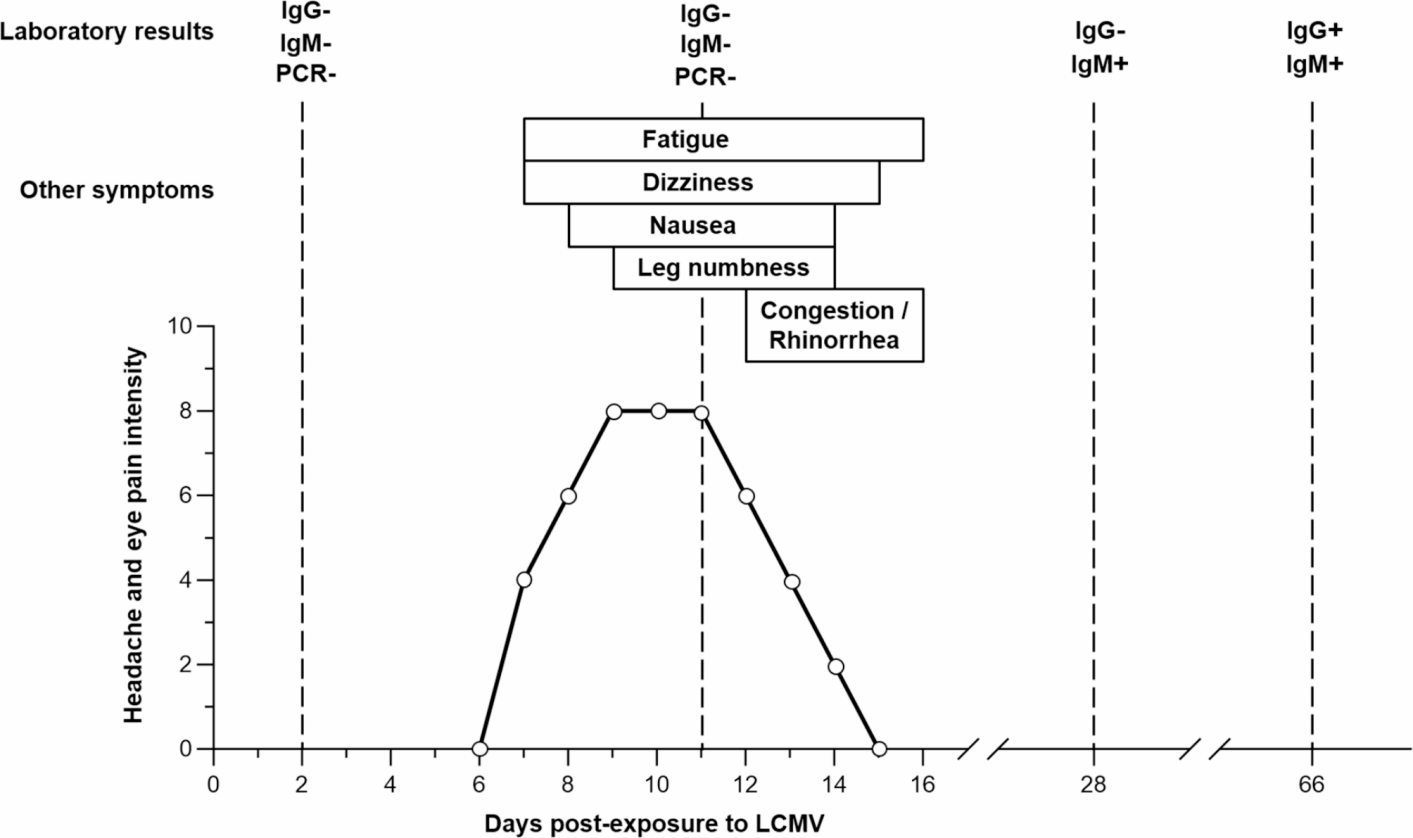
**Timeline of symptoms and blood test results following LCMV exposure**. Symptoms such as headache and eye pain appeared seven days post-infection, with a severity that was highest between day 9 and day 11 post-infection. Other symptoms included fatigue, dizziness, nausea, leg numbness, congestion, and runny nose. All symptoms lasted for less than ten days. Presence of LCMV-specific IgM was first detected at day 28 post-infection and IgG antibodies were detected at day 66 post-infection. PCR testing was only performed at early time points and was negative

At this first visit, two days (D2) post-exposure, physical examination was unremarkable. The LCMV RNA PCR [12] and serology (IgM and IgG by ELISA [13]), performed at the National Microbiology Laboratory (NML; Winnipeg, Canada), were negative. As detailed in Fig. 1, symptoms started on D7. On D10, she went to the ophthalmology department complaining of very intense eye pain, headaches, dizziness, and nausea. No lesions were observed on magnetic resonance imaging (MRI) of the brain, other than the previously mentioned cerebral venous sinus thrombosis sequelae. Due to the mildness of symptoms, a lumbar puncture was not performed. On D11, following the recommendation of the ophthalmologist, the worker returned to the hospital for a follow-up. She had numbness in her left leg, bilateral eye pain with movement, headache, fatigue, dizziness, and nausea, but no vomiting (Fig. 1). Physical examination was unremarkable. A second blood sample was drawn for both LCMV RNA PCR and serology; the results were again negative (Fig. 1). Additional blood tests were performed at D28 and D66. IgM antibodies to LCMV were detected at D28, with a titer of 1:1600. At D66, LCMV-specific IgM antibodies were still present and IgG seroconversion was observed, with a titer of 1:400 for both antibodies. Three months after the incident, full recovery was confirmed, with no residual symptom.

## Discussion and conclusions

In the absence of an alternative explanation for the neurological symptoms, this case supports that LCMV infection via percutaneous injection can cause neurological symptoms in an LCMV naïve individual. It also demonstrates that seroconversion can take more than one month and can occur once all the symptoms have dissipated. We recommend testing for seroconversion at days 30 and 60 post-exposure to confirm the infection status of the individual.

Of interest, the Armstrong strain of LCMV, as well as the Clone-13 variant, is considered as risk group 2 pathogens by the Public Health Agency of Canada (https://health.canada.ca/en/epathogen). In this specific case, the infection occurred in a containment level 2 animal facility, where the standard operating procedures include the use of a second pair of gloves and protective sleeves, as well as clear written procedures regarding the handling of mice on the biosafety cabinets. As the symptoms associated with LCMV infection in humans are relatively mild in immunocompetent individuals (Fig. 1) [2, 14, 15], we argue that the containment level 2 is adequate for performing experiments with the Armstrong strain of LCMV. Moreover, as the risk of self-injection is still present in a containment level 3 facility, the classification of LCMV Armstrong as a level 3 pathogen would not significantly reduce the risk of infection for laboratory workers.

Of note, this case differs from natural infections with LCMV, both in terms of dose and route of administration. The solution prepared for injecting mice contained 2 × 10^5^ plaque-forming units (PFU) per milliliter of LCMV Armstrong and exposure was through a percutaneous wound. Although it is difficult to determine precisely, typical needle-stick injuries can inject between 0.3 µL and 6 µL of volume depending on the gauge and depth of needle exposure [16]. Extrapolating this information to this case leads to an infectious dose of LCMV between 60 and 1200 PFU. The higher viral titer in the inoculum, as well as the percutaneous route of exposure, likely explains the severity of the symptoms. Of interest, in contrast to natural exposure via the respiratory tract, the acute response in this patient was not followed by a second wave of aggravated symptoms.

LCMV is an endemic virus throughout all temperate regions of the world. It is estimated that ~ 10% of wild mice are infected with LCMV, although this number may be higher in some settings [17, 18]. One study reported that approximately 5% of the human population carries antibodies against LCMV [2], but the number of people that have been exposed is likely significantly higher. Studies from the 1960s showed that LCMV was one of the most common causes of aseptic meningitis [19], although the proportion of LCMV meningitis reports has recently declined. This latter point is emphasized by the fact that the NML, which is responsible for all LCMV diagnostics in Canada, only runs ~ 50–60 tests for LCMV per year and only 5 positive cases were found over the last 16 years.

In previous case reports and epidemiological studies of laboratory personnel, LCMV infections were related to the Clone-13 variant or to an unknown strain [14, 15, 20–23]. Common symptoms included fever, severe headaches, flu-like symptoms, vomiting, and, in one case, meningitis [14, 15, 20, 21]. More recently, two cases of LCMV Clone-13 infection through a percutaneous route were reported [7, 8]. The two patients developed neck pain, photophobia, nausea, vomiting, flu-like symptoms, pain in the limbs, and fever [7, 8]. This highlights obvious clinical similarities between LCMV Armstrong and LCMV Clone-13 percutaneous infections, with a possible difference in symptom intensity that could be due to differences in viral doses administered or strain type.

The case presented here is unique given the percutaneous route of exposure and the serological evidence of a primary infection. The previous history of cerebral venous sinus thrombosis makes the differential diagnosis of the patient’s original presentation a little broader, although the absence of neurological symptoms in the years preceding LCMV exposure suggests that a contribution from previous brain lesions is unlikely. Symptoms in a seroconverted individual will likely differ from the symptoms reported here, both in severity and duration. This hypothesis should be confirmed in future reports. This case report offers a framework to investigate and follow patients exposed to LCMV Armstrong, filling a gap in our understanding of LCMV as an endemic pathogen and as a laboratory hazard.

## Data Availability

Data sharing is not applicable to this article as no datasets were generated or analyzed during the current study.

## Abbreviations

LCMV: Lymphocytic choriomeningitis virus
MRI: Magnetic resonance imaging
NML: National Microbiology Laboratory
PFU: Plaque forming units
